# Geometric Morphometrics Reveal Shape Differences in the Toes of Urban Lizards

**DOI:** 10.1093/iob/obac028

**Published:** 2022-08-19

**Authors:** Bailey K Howell, Kristin M Winchell, Travis J Hagey

**Affiliations:** Department of Biological Sciences, Virginia Tech, Blacksburg, VA 24061, USA; Department of Science and Mathematics, Mississippi University for Women, Columbus, MS 39701, USA; Department of Biology, Washington University St. Louis, St. Louis, MO 63130, USA; Ecology and Evolutionary Biology, Princeton University, Princeton, NJ 08544, USA; Department of Science and Mathematics, Mississippi University for Women, Columbus, MS 39701, USA

## Abstract

Urbanization, despite its destructive effects on natural habitats, offers species an opportunity to colonize novel niches. Previous research found that urban *Anolis* lizards in Puerto Rico had increased adhesive toepad area and more ventral toepad scales, traits that are likely adaptive and genetically based. We further investigated these phenotypic changes using geometric morphometrics to measure differences in toe shape, toepad shape, and lamellar morphology. Our results indicate that the increased toepad area of urban *Anolis cristatellus* lizards in Puerto Rico is not simply an isometric increase in toe size. Toes of urban populations exhibit multiple disproportional changes compared to forest lizards, with a larger proportion of the toe length covered in adhesive toepad. In addition, the toepads of urban lizards increase more in length than width. Lastly, lizards in urban populations exhibit both increased number of lamellae as well as increased spacing between individual lamellae. We also observed regional variation, with urban specimens having significantly more disparity, suggesting similar processes of urban adaptation are likely happening in parallel across the island, yet with region-specific idiosyncrasies, possibly generating more variation in toepad morphology across urban specimens as compared to forest specimens. Considering the use of geometric morphometrics, we found that specimen preparation, specifically how flat and straight toes are during imaging, to be an important factor affecting our data, more so than specimen size or any other meaningful morphological variation. In addition, we found that landmark and semilandmark data can be used to directly estimate toepad area, offering the opportunity to streamline future studies. In conclusion, our results highlight the value of considering toepad morphology in more detail beyond adhesive pad area or number of lamellae. Geometric morphometrics tools may be employed to elucidate subtle differences in shape to better allow researchers to connect changes in morphology to ecology and adhesive performance.

## Introduction

As ecosystems change over time, new niches become available for organisms to exploit. This ecological opportunity can lead to speciation and novel ecological communities (Yoder et al. 2010). The process often occurs when organisms colonize isolated uninhabited environments such as islands, lakes, and mountain tops. The evolution of Caribbean *Anolis* lizards provide an incredibly well-studied example. Within each of the Greater Antillean islands, anoles diversified along the same axes of structural and climatic habitat, with species using the same microhabitat evolving convergent morphology including limb length, tail length, body size, head shape, color, and adhesive toepad size (reviewed in Losos 2009). Consequently, trait-environment relationships in anoles are widely considered an evolutionary model for studying ecological speciation, functional morphology, convergence, and adaptation.

There is also suggestion that novel functional traits (i.e., key innovations), rather than island colonization, can drive expansion into previously unoccupied niches leading to increased diversification (Burress & Muñoz 2022). One hypothesized key innovation is adhesive toepads in lizards (Garcia-Porta & Ord 2013; Miller & Stroud 2022). Anole toepads are similar to those of geckos and some arthropods (Irschick et al. 1996; Labonte & Federle 2016). Anole toepads are composed of lamellae, or scansors; specialized scales on the ventral surface covered in a dense array of setae, tiny hair-like projections that generate van der Waals forces enabling these lizards to cling to surfaces (Ruibal & Ernst 1965; Autumn et al. 2002). Previous research has shown that toepad area is positively correlated with cling force, presumably because larger toepads have more setae to engage surfaces at the microscopic level (Irschick et al. 1996; Irschick, Herrel & Vanhooydonck 2006). In addition, species that perch higher in arboreal habitats possess larger toepads with more lamellae, presumably conferring greater clinging ability for life in the treetops (Glossip & Losos 1997; Macrini, Irschick & Losos 2003). Although toepads have likely played an important role in anole specialization, anoles use their toepads in conjunction with their claws to cling to rougher perches (Macrini, Irschick & Losos 2003; Crandell et al. 2014; Naylor & Higham 2019; Yuan, Wake & Wang 2019; Falvey et al. 2020). It has been suggested that perch texture may affect pad performance and hence influence the evolution of toepad area (Winchell et al. 2018b). If a population uses smooth substrates, where claws may be less useful, it may be advantageous to have larger toepads to produce greater traction and reduce falls (Naylor & Higham 2019). Given our strong background knowledge of anole ecomorphology, anoles make a great study system to investigate how a key innovation such as adhesive toepads can be shaped by adaptation to novel environments and drive ecological speciation (Garner et al. 2019).

In the Anthropocene, ubiquitous human impacts have drastically changed the face of the earth. One of the clearest examples of this drastic modification is deforestation and the replacement of wilderness areas with urban environments (Forman 2014). While this has led to the loss and displacement of biodiversity worldwide (McKinney 2002), recent studies have found that urban environments can be a source of ecological opportunity leading to evolutionary change (Johnson & Munshi-South 2017). The crested anole (*Anolis cristatellus*; Duméril and Bibron, 1837) is an urbanophillic species of anole lizard, occupying forest and urban habitats throughout its native range in Puerto Rico (Winchell et al. 2016; Winchell et al. 2018a). Prior research on this species has documented behavioral, ecological, morphological, and physiological shifts between adjacent urban and forest populations within its native range (Winchell et al. 2016; Winchell et al. 2018a, 2018b; Avilés-Rodríguez & Kolbe 2019; Campbell-Staton et al. 2020).

Although responses to and tolerance of urbanization in anoles likely involve a combination of traits (Winchell et al. 2020), two axes of adaptive morphological differentiation stand out as important in urban environments. Urban populations of *A. cristatellus* exhibit heritable morphological shifts towards longer limbs and augmented toepads, that is, larger with more lamellae (Winchell et al. 2016; Winchell et al. 2018b). Previous studies suggest that perch characteristics are important factors in how pad bearing lizards use urban environments (Taylor, Daniels & Johnston 2015; Winchell et al. 2018a). Increases in toepad area and lamella number in urban *A. cristatellus* are presumably adaptations to smoother surfaces more often encountered in urban environments. Winchell et al. (2018b) found that lizards with larger toepads and more lamellae outperformed those with smaller toepads and fewer lamellae on smooth surfaces where claws were likely ineffective. Toepads likely enhance locomotor performance on these surfaces by reducing loss of traction and aid in counteracting the backward pitching effect generated by longer limbs when climbing vertical surfaces (Kolbe et al. 2015; Winchell et al. 2018b; Battles, Irschick & Kolbe 2019; Naylor & Higham 2019). These phenotypic shifts have likely conferred a performance advantage to urban *A. cristatellus*, particularly on smooth vertical substrates such as painted walls and fences common to urban environments (Winchell et al. 2018b). In addition, these patterns of urban adaptation are likely not unique to *A. cristatellus*. Parallel changes have been observed in *Anolis carolinensis* (Cuvier & Voigt 1832). Urban *A. carolinensis* in park-like habitats in Louisiana have larger toepads than those found in nearby forested habitat, an effect the authors hypothesize is due to the use of smoother broadleaf vegetation in anthropogenic environments (Irschick et al. 2005).

**Fig. 1:**
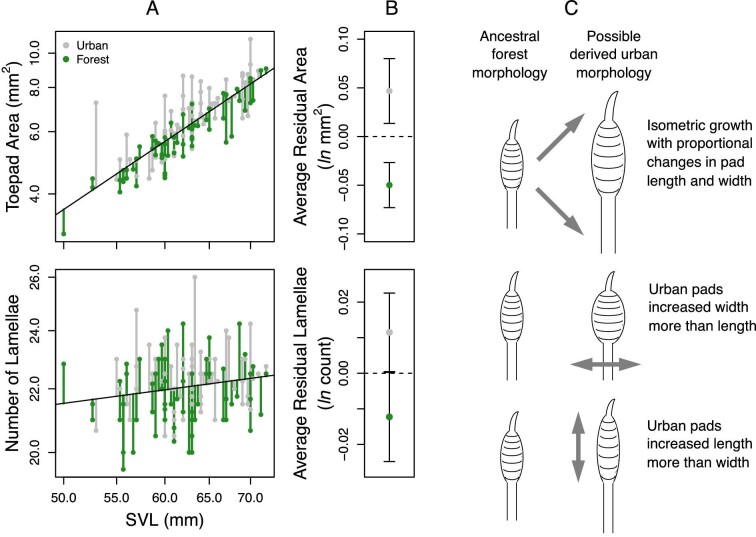
Winchell et al. (2018b) found urban *A. cristatellus* have larger toepads with more lamellae. Using data from Winchell et al. (2018b), we highlight these differences (urban individuals in grey, forest lizards in green) for rear toepad area and lamella counts plotted against SVL (A). Plotting average residual toepad area and lamella counts (with 95% confidence intervals) highlights significant differences between urban and forest lizards (B). Hypothetically, toepad area may change isometrically with no change in overall pad shape (C top illustration) or non-isometrically through a disproportionate increase in toepad width or length (C center and bottom illustrations). Our analyses investigate which of these scenarios best describes previously observed toepad differences between urban and forest populations.

We further investigated how toepads have changed in urban populations of *A. cristatellus*. Using geometric morphometrics, we move beyond simply measuring toepad area and lamella number, or even taking linear measurements of toepad shape, to more precisely and comprehensively quantify how toepad shape has changed in urban anoles. Specifically, we explore how increased toepad area of urban anoles is achieved, whether isometrically, with proportional changes in both pad length and width, or through disproportional changes in toepad width or in length (Fig. 1) resulting in an overall shape change in addition to an increase in size. We also considered how urban anoles fit more lamellae into their large toepads (Fig. 1 data modified from Winchell et al. 2018b), asking how pad size and lamellae organization within the pad has changed in urban populations. As this study represents one of the first to apply geometric morphometrics to toepad morphology, we also conducted a set of analyses to describe our approach. This deeper investigation into toepad morphology, an adaptive trait in urban and non-urban populations of anoles and geckos, will foster future functional morphology and biomechanical studies investigating relationships between toepad size, shape, ecologically relevant performance, and microhabitat use.

## Materials and methods

We captured live animals from the wild and imaged their toepads for subsequent analyses. We used high-dimensional analyses of variance, canonical variance analyses for visualization, descriptive principal component (PC) analyses, and linear morphometric analyses to investigate how toepad shape and lamellar morphology differ in urban anoles. We also conducted analyses generally exploring the use of geometric morphometrics to study toepad shape.

### Field methods

We sampled adult male *A. cristatellus* from 13 sites across Puerto Rico (7 urban and 6 forest sites) in 5 municipalities (San Juan, Arecibo, Aguadilla, Mayagüez, Ponce) between 2012–2016 (Winchell et al. 2016; Winchell et al. 2018b). See table S1 for locality sampling information. Paired urban and forest sites within each municipality were within 10 km of each other. Urban sites were residential neighborhoods and university campuses, characterized by extensive impervious surfaces, minimal tree cover, and abundant anthropogenic structures (e.g., buildings). Forest sites were mature secondary forests, including tropical dry forest (Ponce), and moist forest (San Juan, Arecibo, Aguadilla, Mayagüez). Forest sites had extensive canopy cover and few anthropogenic structures except for walking paths, although some forest sites (e.g., San Juan) were immediately adjacent to urbanization (and so we cannot rule out that lizards do not encounter this habitat at some point during their lifespan).

We captured lizards as encountered using standard methods (extendable pole with floss lasso or hand capture) and did not discriminate between lizards on anthropogenic substrates versus natural substrates. We transported lizards to a field laboratory in each municipality where we imaged rear feet using a flatbed scanner (Epson V300) at 2100–2400 dpi (see Winchell et al. 2016). A single researcher (KMW) measured snout-vent length (SVL) to the nearest 0.5 mm using a transparent ruler. Animals were released after imaging at their location of capture.

### Data collection

**Fig. 2:**
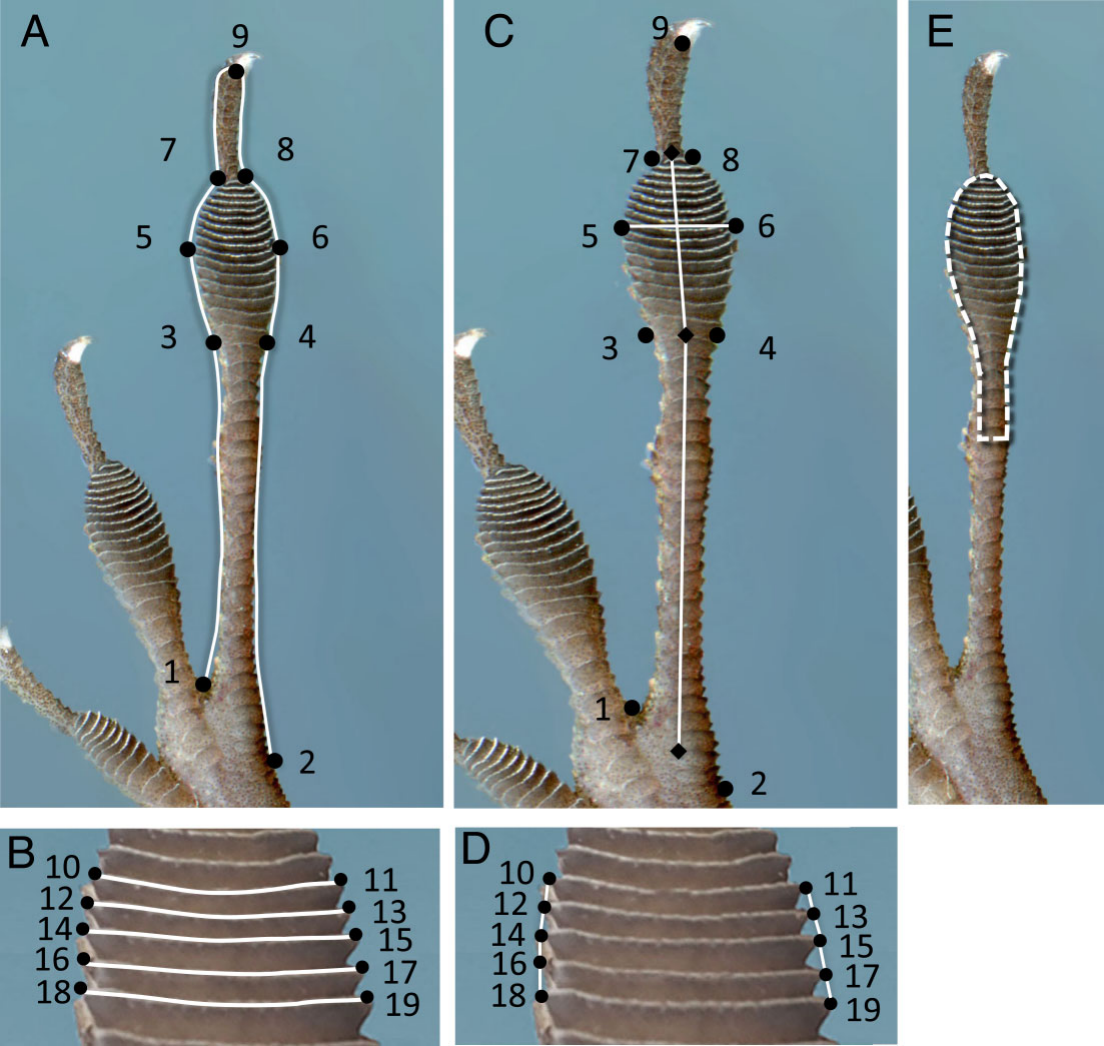
We used geometric morphometrics to investigate changes in toe, toepad, and lamellae shape. Image (A) shows the location of our first nine landmarks and eight curves capturing the shape of the toe and pad. Image (B) illustrates the location of landmarks 10 through 19 and the five curves connecting them to illustrate the size and location of the 5th through 10th lamellae. We also extracted linear measurements of toepad shape. Image (C) illustrates line segments to quantify toepad width, toepad length, and the length of the proximal toe segment. Image (D) illustrates how we quantified average lamellae height based on the distance between consecutive lamellae landmarks. Lastly, we outlined toepad area to the nearest phalangeal joint below the pad to estimate toepad area and counted lamellae within this area (E).

The above sampling represents a large dataset spanning 4 years and including over 1000 lizards (Winchell et al. 2016; Winchell et al. 2018b) from which we selected 13–20 individuals at random (using a random number generator) from each population for our analyses. We included all available individuals for sites with <20 imaged individuals (Table S1). Focusing on the longest rear toes (fourth digit), we decided between each individual's left or right rear toe by choosing the toe of higher quality imaging (i.e., straight and uniformly flattened with no damage). Images of right rear toes were mirrored so that all images were comparable. We focused on the longest rear toe since it has been shown to be important for ecologically relevant performance capabilities in other lizard groups (Zani 2000; Schulte et al. 2004). We developed a novel geometric morphometrics landmarking scheme to capture the entire toe shape, toepad shape, and size and location of the 5th through 10th lamellae (Fig. 2). It is worth noting that geometric morphometric analyses do not explicitly require developmentally homologous landmarks, so long as landmarks are consistently reproducible across specimens. In addition, recent research suggests the distal lamellae of anoles may be developmentally homologous and distinct from more proximal lamellae (Griffing et al. 2022). All images were landmarked by a single researcher (BKH) using tpsDig2 (Rohlf 2010). We used 19 landmarks and 13 curves for each specimen. Curves initially contained 10 semi-landmarks with the first and last semi-landmark of each curve overlapping anchoring landmarks, which were removed in the initial steps of our analyses, resulting in each curve containing eight semi-landmarks.

Landmarks one and two were placed at the base of the toe on the left and right sides where the toe joins the palm, with landmark one at the base of toe three and four and landmark two near toe five. Landmarks three and four were placed on the left and right sides of the toe, respectively, where the proximal end of the toepad begins to widen. Landmarks five and six were placed at the widest left and right points of the toepad. Landmarks seven and eight were placed on the left and right sides of the distal end of the toepad, and landmark nine was placed at the tip of the toe, on the ventral, proximal base of the claw. Landmarks were connected with curves to outline the toe (eight curves total). We also placed landmarks to capture the size and relative location of lamellae. We identified the 5th through 10th lamellae counting distally to proximally, placing landmarks on the left and right sides of each (landmarks 10–19) with a curve outlining the free edge of each lamella. We focused on these specific lamellae as they are large and can be easily traced and therefore are more reliable for comparison. It is worth noting that although the 5th–10th lamellae did not always overlap with the widest part of the toe, they often did. We calculated a millimeter/pixel scale ratio for each image from included scale bars if included. If a scale bar was absent, we used the known scan resolution to calculate a millimeter/pixel scale ratio. Since all specimens were imaged on a flatbed scanner (i.e., there was no variation in the distance from the imaging source), the number of pixels per millimeter in an image is an accurate metric to convert pixel distance to linear distance.

To complement our landmark data, we counted the number of lamellae and measured toepad area for each focal toe using ImageJ with the ObjectJ plugin (Abràmoff, Magalhães & Ram 2004). Deciding how far in the proximal direction to count lamellae and define toepad area can be difficult; previous studies have defined the proximal toepad boundary at either phalanx three or four (Glossip & Losos 1997; Hahn & Köhler 2010) whereas others have relied on lamellae width (Yuan et al. 2020). We defined toepad area to include the dilated area of the toe and the area directly below to the nearest phalangeal toe joint, consistent with Winchell et al. (2016, 2018b) (Fig. 2). We did not include area above the dilated pad area in the distal direction towards the claw. We counted the number of lamellae within this area three times per digit, retaining the consensus number of the three counts (as in Winchell et al. 2016; Winchell et al. 2018b). Toepad area was measured multiple times for a subset of individuals (N = 150) to assess repeatability; we used the average of all area measures per toe in our analyses. Repeatability of toepad area measurements was high (intraclass correlation coefficient: 0.997) and within group variation was far less than among group variation (within: 0.010, among: 2.745). These data were collected by KMW.

### Geometric morphometrics analyses

To process and analyze our data, we used the R studio interface (v. 1.2.5033, RStudio Team 2020) as well as the *geomorph* (v. 3.2.1, Adams & Otarola-Castillo 2013)*, Morpho* (v. 2.8, Schlager 2017), *shape* (v. 1.4.4, Dryden 2021), *pracma* (v. 2.2.9, Borchers 2019), and *car* (v. 3.0–2, Fox & Weisberg 2019) packages. We performed a generalized Procrustes analysis to align our landmark data. This initial step aligns all specimens and removes absolute size. We optimized our semi-landmark locations by minimizing bending energy, a standard approach to optimizing semi-landmark locations. To review our data for errors, we plotted each individual's landmark and semi-landmark locations both before and after alignment (see Supplemental Fig. S1). Outlying individuals were reviewed, and either re-landmarked or replaced when possible if their toes were bent or distorted. This resulted in a final dataset of 246 individuals.

We used a series of complementary analyses to test for statistically significant shape variation and to describe group variation in toepad shape. First, to investigate variation in toepad shape across individuals from urban and forest environments and test the hypothesis of isometric change, we correlated our aligned toepad shape with our categorical habitat variable (urban or forest) using a type-2 sum of squares analysis of variance including an interaction with municipality using the *geomoprh* “procD.lm” function. Given the Procrustes alignment process, if toepads of urban lizards are simply isometrically scaled versions of forest toepads, then there should be no significant aspects of shape that correlate with habitat. We also tested for significant differences in shape disparity between urban and forest lizards using the “morpho.dist” function in *geomorph*, again including municipality in our model. These analyses are designed for high-dimensionality shape data.

In addition to our analyses of variance, we used PC analyses to better understand shape variation in our dataset. To generate our PC data, we used the *geomorph* function “gm.prcomp.” We extracted PCA axes 1, 2, and 3 for each individual and projected toepad shape at the extremes of these three PC axes. We also considered between-group PC analyses (bgPCA) using the *Morpho* R package to visualize shape differences between urban and forest lizards and across municipalities (see Supplement Figs. S4–S5).

**Fig. 3:**
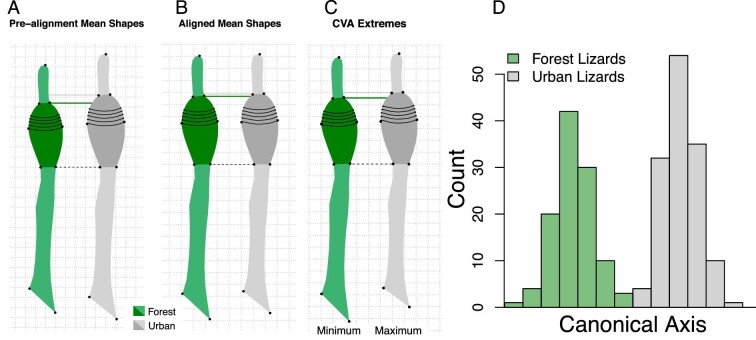
To visualize differences in toepad shape between urban and forest lizards, we plotted (A) the mean shape before Procrustes alignment to visualize absolute size and shape differences between habitat types, and (B) mean shape after Procrustes alignment to visualize only differences in shape. (C) Our CV analyses also produced projected extreme urban and forest shapes representing the maximum and minimum shapes along our CV axis. All displayed shape pairs are vertically aligned along the base of the toepad (dashed black lines). Green horizontal lines demarcate the top of forest toepads while grey horizontal lines demarcate the top of urban pads. Grid increments in A represent 0.5 mm while grid increments represent 0.02 units in Procrustes alignment space (B & C). A histogram of our single CV axis (D) highlights the difference in shape between urban and forest individuals (see Fig S3 for an evaluation of significance).

We visualized shape differences between urban and forest populations and across municipalities (as well as absolute differences in size) with the average pre-alignment and aligned shape of urban and forest lizards (Fig. 3, see Fig. S2 for municipality specific comparisons). To visualize the relationships between shape and habitat category suggested by our above analysis, we used Canonical Variate Analysis (CVA), implemented with the “CVA” function in the *Morpho* R package. Similar to PC analyses, CV analyses also identify axes of variation in highly dimensional data, but instead of identifying the most variable dimensions, a CVA maximizes differences between assigned groups. We note that CV analyses can find axes that partition provided groups by chance. In fact, this is expected given the high dimensionality of geometric morphometrics data. Consequently, we did not consider our resulting one-dimensional CVA as evidence of significant differences in shape between urban and forest individuals (these conclusions were based on our analysis of variance described above). To provide context to our CVA results, we generated a set of CVA results using randomized urban and forest habitat category assignments across individuals. Using 100 randomized datasets, we compared the resulting set of CVA results to our actual CVA results to demonstrate the degree in which a CVA finds axes that separate our data by chance (Fig. S3).

### Linear measurements and analyses

The Procrustes alignment process includes isometric enlarging and shrinking of individual specimens to eliminate the effect of size while maintaining shape. Because of these individual-specific transformations, if our results suggest non-isometric differences in toepad shape between urban and forest lizards, it can be difficult to infer if individuals with larger toepads had wider or longer toepads. As a result, we also extracted linear measurements from unaligned landmarks data (Fig. 2) to investigate differences in toe, toepad, and lamellae proportions. We measured toepad width (distance between landmarks 5 and 6) and toepad length (distance between the midpoint between landmarks 3 and 4 and the midpoint between landmarks 7 and 8). We determined the length of the toe segment from the palm to the proximal base of the pad using the distance between the midpoint of landmarks 1 and 2 and the midpoint of landmarks 3 and 4. Lastly, we measured average lamella height (not in the vertical direction, but in the proximal-distal direction) by averaging together the distances between adjacent edge landmarks 10 and 12, 12 and 14, 14 and 16, 16 and 18, 11 and 13, 13 and 15, 15 and 17, and 17 and 19 (Fig. 2).

If differences between forest and urban lizard toepads were simply isometric differences in size, then linear proportions, such as the ratio of pad width to pad length, would be similar between urban and forest populations. We calculated the natural-log-transformed ratio of toepad width to length for each individual and compared these data between urban and forest lizards using an independent two-group two-tailed *t*-test. This analysis evaluates if urban toepads increased more in width (higher width-to-length ratio) or length (lower width-to-length ratio), or if they are simply isometrically larger (non-significant differences between urban and forest width-to-length ratios). We also investigated how changes in toepad size relate to the rest of the toe using independent two-group two-tailed *t*-tests. We asked if total toe length (natural-log-transformed sum of pad length and proximal toe segment length) differs between urban and forest lizards, which we expect since urban lizards are generally larger. We also asked if the natural-log-transformed ratio of pad length to total toe length differs between urban and forest lizards and if the natural-log-transformed proximal toe length differs between urban and forest lizards to determine if the pad is covering similar proportions of the toe in urban and forest populations.

To complete our investigation using linear measurements, we next focused on lamellae and their relationship with habitat category and the rest of the toe. While previous studies considered toepad area and lamellae counts relative to lizards’ body size, we did not standardize our lamellae data by body size in order to directly compare our lamellae data to other dimensions of toepad size and shape. We are interested in aspects of lamellae morphology regardless of if they are found on large bodied or small bodied individuals. We asked if our data confirm previous studies finding urban lizards have larger toepads and more lamellae via two-tailed *t*-tests using natural log transformed values of area and square root transformed lamellae counts (Whitlock & Schluter 2015). We then asked if lamellae height (in the proximal-distal direction) differs between urban and forest populations, again using a *t*-test with natural log transformed data. To identify potential interactions between toepad area, toepad length, lamellae number, and lamellae height, we used the “Anova” function in the *car* R package to conduct type-2 analyses of variance using generalized linear models. We first asked how area is related to lamellae structure, testing if natural-log-transformed toepad area is correlated with natural-log-transformed lamellae height, square root transformed lamellae count, or the interaction of lamellae height and count. We then considered lamellae height and count, asking if natural-log-transformed lamellae height or square root transformed lamellae count is predicted by natural-log-transformed pad length, habitat category, or their interaction. Lastly, we evaluated the correlation between lamella height and count.

### Methodological analyses

Given our novel approach of applying geometric morphometrics to investigate adhesive toepad shape, we also conducted a series of analyses to further elucidate the use of this method. We evaluated the utility of estimating area using geometric morphometric landmarks compared to two different measurements of toepad area and centroid size. We also evaluated the presence of toepad allometry and how size was captured by our PC axes. These methods and results are described in the supplement.

## Results

### Geometric morphometrics results

Our first statistical comparisons correlating toepad shape with habitat type (urban or forest) and municipality using a Procrustes ANOVA found significant relationships between shape and habitat (*P* = 0.001), municipality (*P* = 0.001), as well as a significant interaction between habitat and municipality (*P* = 0.001). We also found a significant difference in toepad shape variation (shape disparity) between urban and forest lizards after accounting for municipality: urban toepads were 18% more variable than forest toepads (*P* = 0.026). To visualize these results, we plotted the mean shape of urban and forest lizard toepads before and after Procrustes alignment (Fig. 3) as well as mean shapes from individual municipalities (Fig. S2). We observed striking difference in absolute toepad size in urban lizards (Fig. 3A), with larger toepads and longer toes. After specimens were scaled during our Procrustes alignment process (Fig. 3B), subtle differences in shape can be seen. Urban pads still appear larger, with lamellae 5–10 shifted distally, while the proximal section of the toe, from the base of the pad to the palm of the foot, appears shorter.

We further visualize these differences in our CVA projections (Fig. 3C). Since we only had two habitat categories, our results produced a single CV axis (Fig. 3) that captured 8.6% of the variation and in which urban and forest individuals are clearly separated. This analysis separated urban and forest individuals more than expected by our null dataset, generated by 100 simulated CVAs (Fig. S3). Specifically, our randomized CV analyses found axes that separated randomly assigned categories by an average distance of 4.28 canonical variate score units (standard deviation: 0.47), with the largest observed separation among all 100 simulations of 5.81 units. Our actual urban and forest groups were separated by 5.89 units, suggesting the differences in shape between urban and forest lizards characterized by the CVA are meaningful. We projected toepad shape along our single identified CV axis, generating shapes that represent a projected urban toepad shape and a forest toepad shape. Similar to our estimates of urban and forest lizard mean toepad shapes, our projected CV axis shapes also suggest urban lizards have disproportionately longer toepads that make up a larger proportion of the total toe length with lamellae shifted distally (Fig. 3).

**Fig. 4:**
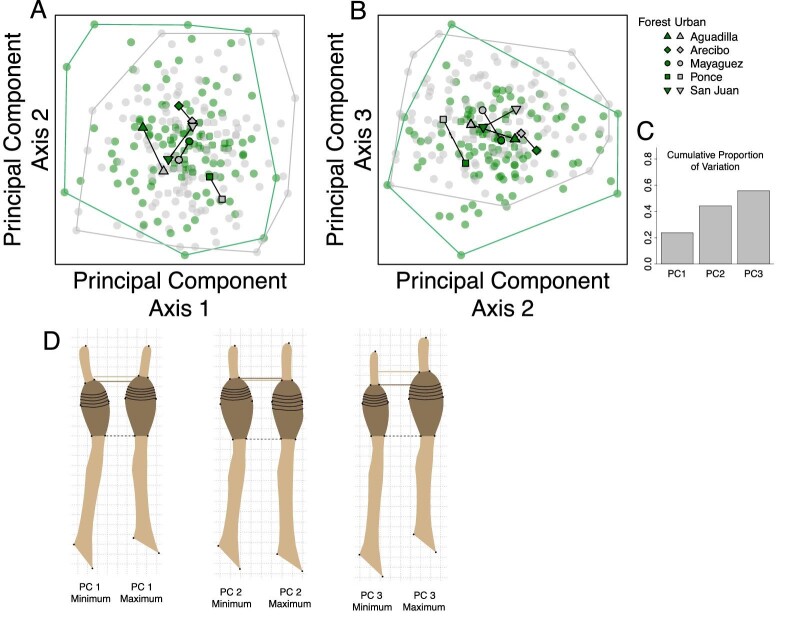
Principal component analysis of toepad shape showing the first three principal components (A and B), with each lizard represented by semitransparent points (urban individuals in grey, forest individuals in green). Mean municipality values for each principal component are illustrated by paired shapes with black lines connecting forest and urban means within each municipality (Aguadilla: triangles, Arecibo: diamonds, Mayagüez: circles, Ponce: squares, San Juan: inverted triangles). Histogram (C) visualizes the proportion of the total variation in our dataset captured by each of our first three PC axes. Toepad illustrations (D) represent shape projections for each PC axis, vertically aligned along the toepad base (black dotted line) with additional horizonal lines highlighting the distal end of each projected toepad shape.

We next investigated general patterns in our shape data using PCs analyses (Fig. 4). To provide context to our PC axes, we produced shape estimates of the maximum and minimum axes extremes (Fig. 4D). PC 1 explained 23.8% of the variation in our dataset, PC 2 explained 20.5%, and PC 3 explained 11.5% (Fig. 4C). PC 1 appears to primarily capture aspects of how straight each toe was at the time of imaging, and specifically whether toes bent to the left (lower PC 1 values) or right (higher PC 1 values). In addition, PC 1 also captures aspects of relative toe and toepad proportions. Individuals lower on PC1 have longer proximal toe segments (distance from the base of toe to the base of the pad) and toepads shorter in length, whereas individuals with higher values on PC 1 have proportionally longer pads and/or shorter proximal toe segments. Our projections of PC 2 capture aspects of lamellae location and lamellae height. Lamellae 5–10 of individuals lower in PC 2 are more distally located and are closer together (narrower) suggesting these individuals may have more total lamellae. Conversely, lamellae 5–10 of individuals on the upper end of PC 2 sit lower on the toe and are more widely spaced, suggesting these individuals may have fewer lamellae. PC 2 also appears to capture aspects of toepad length and distal toe segment length (the distance of the claw from the pad), with specimens lower on PC 2 having a longer toepad and shorter distal segment, whereas specimens higher on PC 2 have shorter pads and longer distal segments. Lastly, PC 3 captures toepad size, including both length and width. Individuals higher on PC 3 have proportionally wider toepads that make up a larger proportion of the toe's total length. Our between-group PC analysis using habitat category (urban or forest) reinforced these results, suggesting urban lizards have proportionally larger pads and shorter proximal toe segments, with lamellae shifted distally (Fig. S4). Our results from the bgPCA investigating variation across municipality suggested strong differences between municipality with similar patterns as seen in our PCA (Fig. 4), with our bgPC axis 1 capturing aspects of lamellae as seen in PC 2, and bgPC axis 2 capturing subtle aspects of toepad size (Fig. S5).

### Linear measurements

**Fig. 5:**
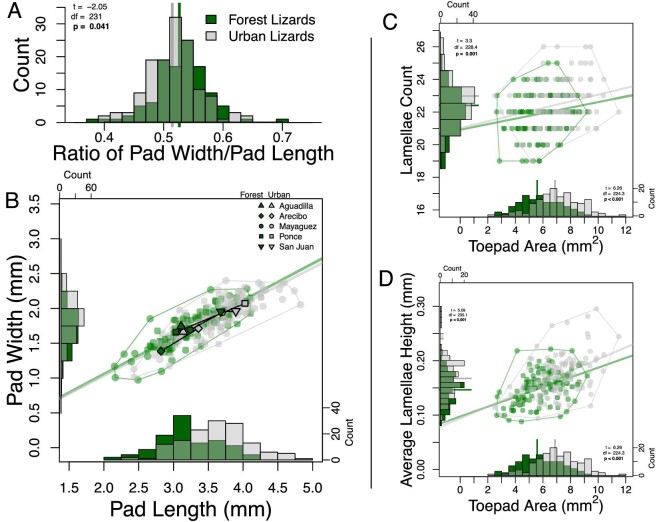
Linear and area measurements help us better understand shape differences. Histogram (A) visualizes the difference in the mean ratio of width/length between urban and forest lizards. Scatterplot (B) illustrates the same data, comparing pad width versus length in millimeters. The slopes of the fitted lines through the origin represent the mean ratios for urban and forest lizards across our dataset. The grey urban line's location below the green forest line illustrates the lower width/length ratio in urban lizards, suggesting urban lizards gain toepad area via larger increases in length than width. We also include histograms of toepad width and length along the X and Y axes to better illustrate differences between urban and forest lizards and the larger increase in pad length compared to width. Mean values for each municipality are plotted with point shape indicating municipality and black lines connecting each intra-municipality urban and forest pair. Similar municipality-specific scatter plots can be seen in Fig. S6. We also compared toepad area, lamellae count, and lamellae height between urban and forest lizards (C and D). We plotted these relationships as scatter plots with embedded histograms for each axis. We included best fit lines not forced through the origin in our scatter plots for both forest and urban populations. In all plots urban individuals are in grey and forest are in green.

We further investigated our hypothesis of isometric change with a series of linear measurements extracted from our unaligned landmark data. The toepads of urban lizards had an average width of 1.86 mm (sd = 0.28) and an average length of 3.61 mm (sd = 0.47). Forest lizards’ toepads had an average width of 1.69 mm (sd = 0.27) and an average length of 3.20 mm (sd = 0.43). The toepads of urban lizards were on average 10.06% wider than forest lizards, but also 12.7% longer. The mean width to length ratios of toepads differed between urban and forest lizards (two-sided *t*-test, t = −2.05, df = 231, *P* = 0.04): urban lizards have a smaller mean width to length ratio (0.514, 95% CI: 0.507–0.522) compared to forest lizards (0.527, 95% CI: 0.518–0.535) (Fig. 5). These results suggest that the larger toepad size of urban lizards is not isometric and that urban toepads increase in length more than width.

**Fig. 6:**
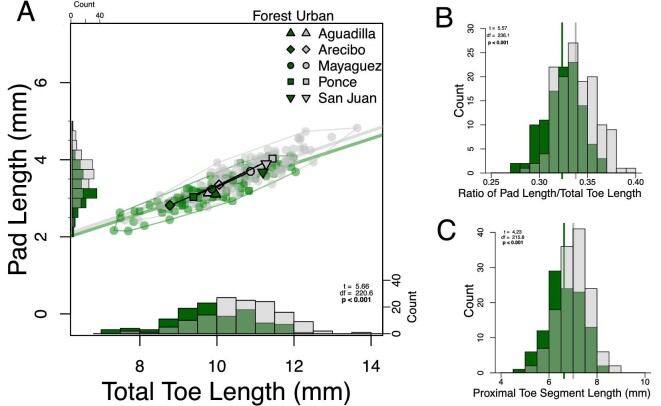
To visualize differences in lizard toe proportions, we plotted the total toe lengths of urban (grey) and forest (green) lizards versus the length of only the pad (A). We highlight the significantly different ratio of pad length to total toe length between urban and forest lizards using green and grey best-fit lines forced through the origin. Histograms of the pad length/total toe length ratios (B) and the proximal toe segment length (C) include vertical lines representing urban and forest population mean values.

We also used our linear measurements to consider how the proportions of the overall toe, and not just the pad, differ between urban and forest lizards. Specifically, we asked how increases in toepad length may also be related to changes in overall toe length and the length of the proximal toe segment (Fig. 6 and Fig. S6). We found that total toe length is longer in urban lizards (Fig. 6; two-sided *t*-tests, t = 5.7, df = 220.7, *P* < 0.001) which is not surprising given that urban lizards are often larger and tend to generally have longer limbs (including the metatarsal and first phalanx on toe IV; Winchell et al. 2016). Forest lizards have a mean total toe length of 9.8 mm (95% CI: 9.60–10.02 mm), while urban lizards have a mean total length of 10.59 mm (95% CI: 10.42–10.78). We next compared the mean ratio of pad length over total toe length between urban and forest lizards (Fig. 6) and found non-isometric differences between urban and forest lizards. The total toe lengths of urban lizards were comprised disproportionally more toepad (t = 5.6, df = 236, *P* < 0.001), covering 1.41% more of their toe lengthwise compared to forest lizards. Urban lizards had mean pad length to total toe length ratio of 0.338 (95% CI: 0.334–0.341) while forest lizards had a mean pad length to total toe length ratio of 0.324 (95% CI: 0.32–0.327). We observed municipality-specific differences in this relationship (Fig. S6). Lizards in Aguadilla and San Juan exhibited minimal differences between urban and forest lizards, whereas more pronounced differences were observed in Arecibo, Mayagüez, and Ponce. Lastly, we investigated change in the proximal toe segment, the distance between the base of the pad and the base of the toe (Fig. 6). We found significant differences in the mean proximal toe length (t = 4.2, df = 215.8, *P* < 0.001), with urban lizards having significantly longer proximal toe segments (urban = 7.01mm 95%CI 6.90–7.12, forest = 6.63 95%CI 6.49–6.76). Together these results suggest that although urban lizards have longer toes and larger toepads in an absolute sense compared to forest lizards, these differences are not isometric. Urban lizards have disproportionately longer toepads, and their toepads cover disproportionately more of their toe.

In addition to investigating toe and toepad differences between urban and forest lizards, we also used the linear measurements derived from our landmarks to compare aspects of lamellar morphology (Fig. 5). As reported in previous studies (Winchell et al. 2016; Winchell et al. 2018b), urban lizards had significantly larger toepads (t = 6.26, df = 224.3, and *P* < 0.001) with significantly more lamellae (t = 3.30, df = 228.4, *P* = 0.001). Average urban lizard toepads were 6.87 mm^2^ (95% CI: 6.58–7.16) whereas average forest lizard toepads were 5.57 mm^2^ (95%CI: 5.29–5.86). Urban lizard toepads also had an average of 22.44 lamellae (95%CI: 22.22–22.67), whereas forest lizards had 21.87 lamellae (95%CI: 21.61–22.13). In addition to these confirmatory results, we also found urban lizards to have significantly more distance between their lamellae (t = 5.19, df = 244. 0, *P* < 0.001). Urban lizards had a mean lamellae height of 0.17 mm (95% CI ± 0.006) and forest lizards had an average of 0.15 mm (95% CI ± 0.006).

Through the above analyses, we noted many municipality-specific differences in toepad shape, linear measurements, and lamellar morphology. In Aguadilla, we did not observe substantial differences between urban and forest specimens in toe or pad proportions (Fig. S2, S6), but we did observe distal shifts in the lamellae (Fig. S2 and S5) and an absence of low-lamellae count individuals in urban populations, resulting in an increase in total average lamellae but no change in lamellae height (Fig. S6). Forest individuals from Arecibo had some of the smallest toes across the five municipalities, with urban individuals having much larger toes (Fig. S2) and pads (Fig. S6). The pads of urban individuals were disproportionately larger with shorter proximal and distal toe segments (Fig. S2, S6). The pads of urban individuals increased in width more than length (Fig. 5 and S6) contrary to other municipalities and the overall pattern we observed. Urban individuals also had more and wider lamellae (Fig. S6). In our Mayagüez municipality, urban lizards again had absolutely larger toes with weak relative differences between urban and forest individuals (Fig. 3). We did observe a slight increase in urban lizards’ pad proportion of the toe (Fig. S2, S5, S6), although the pad itself appears to be increasing in size isometrically (Fig. 5, S6) with equal increases in length and width (Fig. S6). Urban lizards also had more lamellae and wider lamellae (Fig. S6). The toes of forest lizards in Ponce were also very small and here we observed the biggest absolute difference in toe size between urban and forest, with urban toes being much larger. We also found strong relative differences in pad-toe proportions and a distal shift in lamellae (Fig. S2, S5, S6). Urban lizards had a clear increase in pad length more than width (Fig. 5). Although forest lizards already had a high lamellae count, urban lizards increased in pad area, lamellae count, and slightly in lamellae height (Fig. S6). Lastly, forest lizards from our San Juan locality had some of the largest pads when compared with other forest lizards, with subtle differences in absolute size in urban individuals. The toes of urban lizards had disproportionately more pad and shorter proximal toe segments (Fig. 3). Considering shape, urban and forest lizards were similar with urban lizards having slightly longer pads and shorter proximal toe segments (Fig. S2, S5, S6) with a subtle shift in pad length more than width (Fig. 5). Urban lizards also had wider lamellae (Fig. S6).

Finally, we investigated relationships between pad area, lamellae height, and lamellae count. We found both lamellae count (type-2 analyses of variance; *P* < 0.001) and height (*P* < 0.001) were positively correlated with toepad area, but the interaction was not significant (*P* = 0.65, see Fig. 5). Lastly, we tested the relationship between lamellae count and height, finding it to be not significant (*P* = 0.22). These results varied across municipalities (Fig. S6): Aguadilla varied little between urban and forest individuals, whereas urban lizards from Arecibo, Mayagüez, and Ponce all increased in toepad area, lamellae count, and lamellae height. Interestingly, San Juan appeared to increase primarily in lamellae height, with minor changes in area and count. Similarly, we considered how toepad length relates to lamellae characteristics (Fig. S7). We found that lamellae height was positively related with toepad length (ANOVA, *P* < 0.001), weakly correlated with habitat (*P* = 0.066), with no significant interaction between habitat category and toepad length (*P* = 0.58). Our analyses of lamellae count yielded similar results, with pad length showing a significant positive relationship (*P* < 0.001), habitat category again not quite being significant (*P* = 0.09) and no significant interaction (*P* = 0.5).

Together these results suggest urban lizards are generally increasing all aspects of their toepads and lamellar morphology by increasing toepad area, lamellae count, and lamellae height, with longer pads also supporting more space between lamellae. Interestingly, these results do not support the idea of a trade-off between lamellae height and lamellae number, but instead suggest increases in toe pad area, specifically toepad length, likely allow increases in both lamellae count and lamellae height.

## Discussion

Previous studies have found that urban populations of *A. cristatellus* in Puerto Rico have larger adhesive toepads and more lamellae when compared to their forest counterparts (Winchell et al. 2016, 2018b). We asked how these populations are developing larger pads, either via isometric changes to the toe or through disproportional changes to their toe and toepad morphology. With the use of geometric morphometrics having never been applied to adhesive toepads, we also explored some of the properties of this approach. Our results reinforce previous findings that urban populations of *A. cristatellus* have significantly different toes and toepads than those of forest lizards (Winchell et al. 2016; Winchell et al. 2018b). We found that, in an absolute sense, the toes of urban lizards are longer with larger pads (Figs. 3, 5, 6) and that these differences are not isometric. When only considering differences in shape, we found that urban lizards’ toes are composed of relatively more pad, (i.e., longer pads and shorter proximal toe segments; Figs. 3, S4, 6). In addition, the pads of urban lizards are disproportionately longer, relative to their increase in width (Fig. 5). These results suggest adaptation to urban microhabitats has changed not only the relative size and proportions of urban lizards’ toepads but aspects of the entire toe. We discuss the potential implications of these findings with respect to urban adaptation as well as more methodological implications in the following sections.

### Urban adaptation

Urban lizards are exposed to different selection pressures than those in forested areas, and the observed increase in overall toe length we found in urban lizards could be driven by functional demands. The fourth toe of the hindlimbs in lizards is related to locomotion, producing propulsion forces during sprinting (Irschick & Jayne 1999; Russel & Bels 2001). Previous studies have shown that urban areas have greater habitat openness (Winchell et al. 2016; Winchell et al. 2018a) imposing functional demands to increase sprint speed which could lead to increases in total length of the fourth toe. Similarly, changes in overall toe length, even if not directly influencing sprint speeds, could be related to increases in hindlimb length, selected for via the use of wider perches, through indirect selection if these traits are functionally or developmentally integrated as some evidence seems to suggest (Spezzano & Jayne 2004; Foster & Higham 2012; Poe et al. 2021). Related but distinct is the possibility that as lizards expand into urban areas they are presented with ecological opportunity. This explanation would be consistent with the increased disparity in the toe shape of urban lizards we observed, however, this greater disparity could also be attributed to greater heterogeneity in urban selection pressures that are imposed in different municipalities (Yoder et al. 2010). Although, we were not able to explicitly address these potential alternatives, they are testable hypotheses that should be considered in future studies.

In addition to changes in absolute toe length, we also found that the toe's length is comprised of more padded area and less proximal toe length in urban lizards. The increases in pad area (via increases in pad length) are not surprising, as this presumably would allow the lizards to climb and cling more easily to the smoother perches common in urban areas as the claw becomes less effective (Winchell et al. 2016, Falvey et al. 2020). Potentially, increasing pad area through increasing length (rather than width) could optimize both functional demands that might be placed on *A. cristatellus* in locomotion both on horizontal (sprinting) and vertical substrates (climbing and clinging) (Simon et al. 2022). However, increases in pad length could also be the result of indirect selection on increases in hindlimb length or total toe length. Decreases in the relative proportions of proximal toe lengths might represent a reduction in phalanges that can be seen in other pad-bearing species of lizards (Zhuang et al. 2019). Russell 1977 suggested that the reduction of more proximal phalanges placed the more distal phalanges in a position that better optimized usage of the claw, which has been shown to work synergistically with toepads to produce cling force in pad-bearing lizards (Crandell et al. 2014; Naylor & Higham 2019; Yuan, Wake & Wang 2019; Falvey et al. 2020). Future studies should consider how toe anatomy changes between urban and forest lizards and if this is related to function or performance, particularly how the claw, pad, and proximal phalanges interact and how this impacts cling force as lizards adapt to using smoother substrates.

While previous studies found that urban lizards have more lamellae on their toepads (Winchell et al. 2016), our results highlight that the lamellae of urban lizards tend to be more distantly interspaced (defined as lamellae height), with lamellae 5–10 more distally located on the toe. This spacing and shift in relative location of lamellae is likely how urban lizards fit more lamellae into the toepad (Figs. 3,7,10). One might expect a negative relationship between lamellae height and lamellae number with the assumption that more lamellae necessitate more tightly packed lamellae, but we found no such trade off. Urban individuals had increases in both lamellae height and count along with pad area. Few studies have considered toepad morphology beyond area and number of lamellae although Macrini, Irschick, and Losos (2003) and Yuan, Wake, and Wang (2019) both found interesting patterns involving toe pad dimensions (especially width), lamellae number, claw morphology, and perch height or diameter, as well as differences between Caribbean and mainland anoles species. These studies suggest pad dimensions and intra-pad lamellae configuration likely influences performance, although the details of this relationship, and how these findings translate at the intraspecific level, remain unclear.

Our results also repeatedly found differences across municipalities, suggesting urban adaptation in toepad shape is not occurring identically across sites in Puerto Rico. This idiosyncratic pattern of parallel adaptation may explain the significant increase in morphological disparity we observed in urban specimens. Although our results confirm a general increase in toepad size and number of lamellae in urban populations, these morphological shifts appear to be occurring in slightly different ways in different populations, similar to previous studies (Winchell et al. 2016). The idiosyncratic variation we saw across municipalities may be examples of plasticity, local adaptation, founder effects, or possibly our modest sample sizes. Genetic analyses suggest that across the island of Puerto Rico there is significant regional differentiation among *A. cristatellus* (Reynolds et al. 2017), including substantial differentiation associated with environmental variation (Wogan et al. 2020). Thus, the standing genetic variation in populations likely varies across municipalities, potentially influencing adaptive outcomes. Moreover, among the populations included in this study, gene flow is highly restricted across municipalities but not between urban and forest sites within each municipality (Winchell et al. 2016, Campbell-Staton et al. 2021). In urban-forest pairs where gene flow is elevated, adaptive responses may be constrained. Notably, the urban and forest populations in Ponce were the most genetically differentiated as well as the furthest apart geographically (Winchell et al. 2016) compared to the other municipalities, possibly explaining why we observed such pronounced morphological differences between urban and forest lizards in this and in previous (Winchell et al. 2016) studies. It's also possible that with larger sample sizes these differences would become more pronounced and consistent across municipalities.

### Methodological results

Our analyses exploring the use of geometric morphometrics to study toepad shape produced a set of important findings and highlighted some considerations for future studies (see supplement). Our PCs analyses (Fig. 4, S9) and the major axes of shape variation in our dataset highlight how specimen preparation can influence a dataset. We recommend future studies planning to measure toe or toepad shape using geometric morphometrics assure that their toes are straight during imaging, otherwise this variation in shape is likely to dominate their dataset. It was not until after specimen condition had been accounted for in PC1 that we saw interesting morphological differences regarding toe proportions, pad proportions, and lamellae location and height differences in our dataset.

We also found specimen size to have an influence on toepad shape, even after size had been removed by Procrustes alignment (Fig. S8 E). When specimen size is defined by toepad area, we see larger specimens having enlarged pad areas, shorter proximal toe sections, and distally shifted lamellae. When specimen size is defined by centroid size, we also see correlations with shape, with centroid size being inflated in specimens with longer toepads (Fig. S8 B). This means that centroid size is likely a poor proxy for toepad area, since short wide toepads will have smaller centroid sizes than long skinny toepads, even with similar areas. Despite this evidence of specimen size and shape being connected in our dataset, our PC analyses did not capture this relationship as expected (Fig. S9). Regardless of how size was defined (as SVL, pad area, or centroid size), it appears PC 2 and PC 3 were capturing aspects of size (Fig. 4), not PC1. This again reinforces the importance of sample preparation during imaging.

Our results also highlight the importance of how toepad size and lamellae count are measured, specifically how proximally on the toe one measures area and lamellae. We found that both centroid size and landmark-based estimates of area can serve as proxies for pad area, although centroid size is affected by specimen compactness (Fig. S8), so it may not always be a great representation of size depending on the specific question being asked and the location of the landmarks for a given study. Depending on the landmark scheme a study is using, the area enclosed by a chosen set of landmarks and semi-landmarks can serve as a proxy for area, or serve as a direct measurement of area, eliminating the need to independently measure area via programs like ImageJ. Lastly, our results highlight how landmarks outlining only a few select lamellae could be informative regarding lamellae count, with distal shifts of our five focal lamellae often indicating more lamellae being present on an individual's toepad.

## Conclusions

Our results highlight how microhabitat differences between urban and forest environments are likely driving the evolution of lizard toepad morphology in subtle ways, changing the toe, pad, and lamellae proportions of urban lizards. Specifically urban populations appear to be moving towards morphologies that exaggerate toepad and lamellae related traits, generally fitting more lamellae that are more distally spaced into larger, and disproportionally longer pads, with a larger proportion of the entire toe composed of pad. This process is likely happening repeatedly and independently at urban locations across Puerto Rico, with subtle differences possibly attributable to variation in the amount of time urban and forest populations have been diverging, the extent of population connectivity, or differences in the direction and/or magnitude of selection urban environments impose within each municipality, resulting in urban specimens having more disparate toepads as compared to forest specimens. Our results suggesting urban lizards having longer toes invites questions regarding how an increase in toe length may be related to phalanx kinematics during gripping and previously observed increases in overall limb length in urban populations and how these differences interact with sprint speed and locomotion kinematics. Lastly, while we did not explicitly consider differences in the distal toe segment, the length from the distal end of the toepad to the claw, we did observe differences between municipalities. Future studies may investigate how distal toe segment length above pad influences the use of the claw and how it may be related to the use of smoother urban surfaces (Falvey et al. 2020). Our results also demonstrate that geometric morphometrics is a viable tool for studying adhesive toepad morphology and open a variety of exciting new research directions. In conclusion, our study furthers our knowledge of adaptive toepad morphology, moving beyond simply measuring pad area and lamellae number to gain a more detailed understanding of how adhesive toepads vary and how populations adapt to changing microhabitats with exciting implications for the biomechanics and performance of toepads and the entire locomotor system of pad bearing lizards.

## Supplementary Material

obac028_Supplemental_Files

## Data Availability

Data and supplemental information/analyses will be available from the Zenodo Digital Repository: https://doi.org/10.5281/zenodo.5495594 and on GitHub: bkhowell/UrbanToepadShape.
