# Supplementary material for: Geometric Morphometrics Reveal Shape Differences in the Toes of Urban Lizards
Source: Integr Org Biol. 2022 Aug 19;4(1):obac028. doi: 10.1093/iob/obac028 (PMC9391197; doi:10.1093/iob/obac028)
Supplement: obac028_Supplemental_Files [file obac028_supplemental_files.zip › Howell_Supplement_Sept9.pdf]

Geometric Morphometrics Reveal Shape Differences in the Toes of Urban Lizards

Supplemental Figures and Tables

Files included in this supplement

Analysis files

- *Howell PR anole shape.tps* – Geometric morphometrics TPS file containing shape data collected by author #1
- *Howell PR anole semilandmark slider file.txt* – Text file containing semilandmark information required for geometric morphometrics analyses
- *Howell Field Data.txt* – Text file containing locality and morphological data collected by author #2 in the field
- *Howell Pad Area Subset.txt* – Text file containing subset of toepad area data measured by author #3

R code files

- *Howell PR Preliminary Data Setup.R* – Initial R code to setup data and perform data quality checks
- *Howell PR Geometric Morphometrics Analyses.R* – R code to conduct geometric morphometrics related analyses and produce results plots
- *Howell PR Linear Analyses.R* – R code to conduct analyses related to our linear morphometrics analyses
- *Howell PR Methodolodical Analyses.R* – R code to conduct analyses investigating the utility of geometric morphometrics when applied to toepads

Geometric Morphometrics Reveal Shape Differences in the Toes of Urban Lizards

Supplemental Figures and Tables

Table S1

We sampled up to 20 individuals from 13 forest or urban sites from 5 different Puerto Rician Municipalities.

Sample Sizes	
Aguadilla	
Forest Site	13
Urban Site	16
Arecibo	
Forest Site	17
Urban Site A	20
Urban Site B	20
Mayaguez	
Forest Site A	20
Forest Site B	20
Urban Site A	20
Urban Site B	20
Ponce	
Forest Site	20
Urban Site	20
San Juan	
Forest Site	20
Urban Site	20
Total	246

<u>Figure S1</u>

We developed custom a R function to plot the landmark locations of each specimen before and after our Procrustes alignments for quality control purposes. We highlight an example plot for individual “KMW_011_RR”. Each landmark is labeled, and each curve is given a unique color and arrow representing the order of the curves (colors) and the order of the landmarks and semilandmarks within each curve (the arrow). Each curve starts and stops at anchor landmarks with eight semilandmarks in between. The unique colors and arrows of each curve allow us to quickly visualize if curves, landmarks, or semilandmarks are out of order.

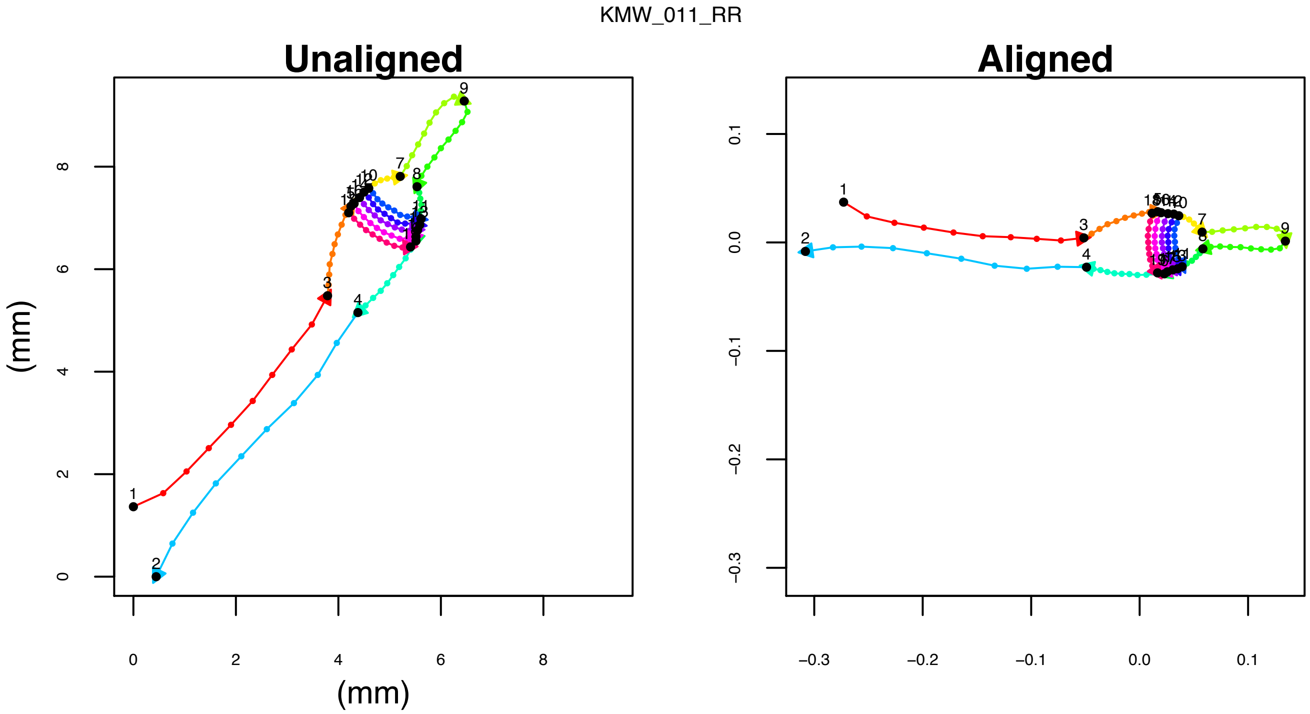

<u>Figure S2</u>

Given our results suggesting a significant interaction between habitat category and municipality affecting toepad shape, we plotted forest (green) and urban (grey) mean toepad shape for each municipality (A). We included the mean shape before Procrustes alignment (left pair of shapes) to visualize absolute size and shape differences between habitat types, and mean shape after Procrustes alignment (right pair of shapes) to visualize only differences in shape. Shape pairs were vertically aligned along the base of the toepad (landmarks 3 and 4, dashed black lines). Green horizontal lines demarcate the top of the mean forest toepad while grey horizontal lines demarcate the top of the mean urban pads. We also included a background grid to also help visualize differences in toepad length and width. The grid represents increments of 0.5 mm (left pair of images) and 0.02 units (right pair of images). We also included plots of PC1 vs PC2 (B left plots) and PC2 vs PC3 (B right plots) limited to individuals from each municipality to help visualize the morphological variation within and across municipalities, again using green to illustrate forest individuals and grey for urban individuals as well as mean forest and mean urban PC values (triangles) connected with a line segment in each plot. The X and Y axes are consistent across all similar PC plots.

Geometric Morphometrics Reveal Shape Differences in the Toes of Urban Lizards

Supplemental Figures and Tables

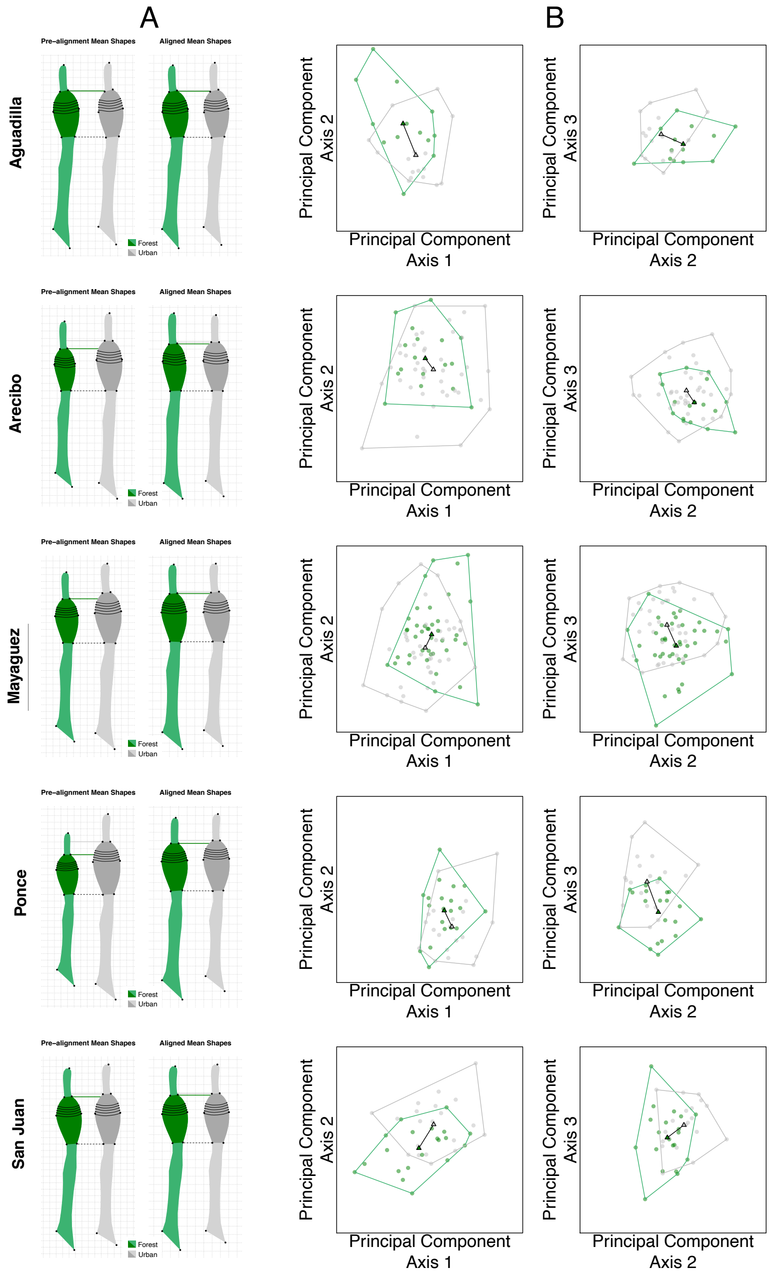

<u>Figure S3</u>

To better visualize the differences between urban and forest lizard toepad shape, we used a CVA. When using high-dimensional data, a CVA can still find axes that separates data by chance. As a result, we conducted 100 CV analyses using our aligned toepad shape data with habitat categories randomly assigned to each individual. Viewing our data as the variation across histograms, the shaded areas represent two standard deviations around the average count value for each bin along the x-axis. Solid lines represent our actual CVA results. While our randomized analyses did typically find bimodal histograms by chance, they typically overlapped more than we observed in our CVA using actual data.

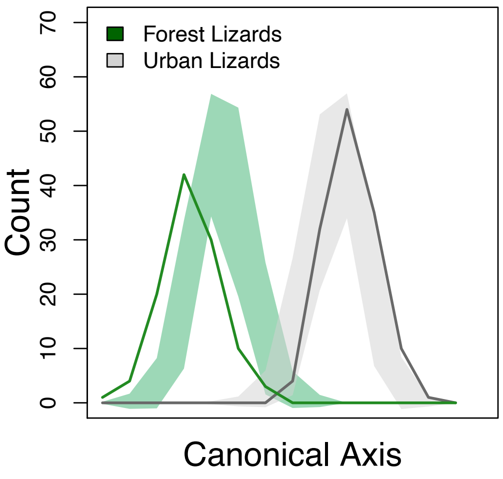

Geometric Morphometrics Reveal Shape Differences in the Toes of Urban Lizards

Supplemental Figures and Tables

<u>Figure S4</u>

Between group PCA identify dimensions that capture the most variation across means of assigned groups. Our first bgPC analysis used habitat category (urban or forest) as assigned groups. It is worth nothing that between-group PCA analyses can be negatively affected by small or uneven samples sizes, although we believe this had minimal effects on our results (Bookstein 2019).

Plotted as a histogram with forest lizards as green bars and urban lizards as grey bars (note the transparent aspects of overlapping bars), our bgPCA axis captured 4.0% of the variation in our dataset (A). We also included shape projections of the lower and upper extremes of our bgPCA axis. (B) The predicted shape represents a shape along the bgPC axis two standard deviations above or below the overall mean. Similar to our other methods of visualizing shape differences, our projected bgPCA shapes again found urban lizards to have proportionally larger pads and shorter proximal toe segments, and lamellae shifted distally.

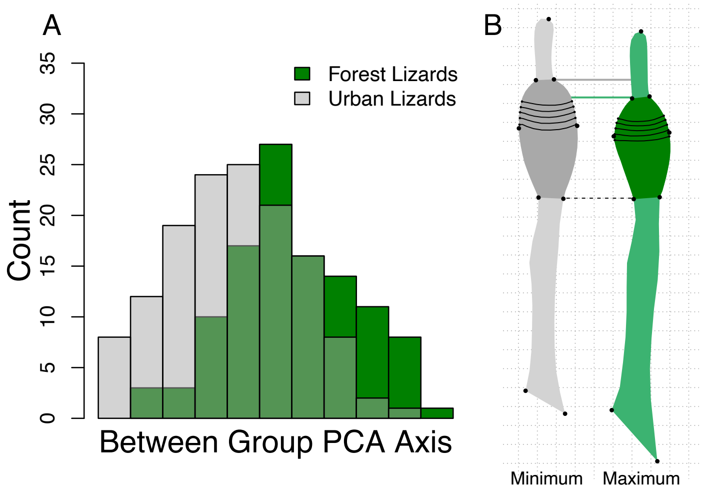

<u>Figure S5</u>

We also conducted a between-group PC analysis considering municipality highlighting strong differences across municipalities. We plotted urban and forest means for each municipality on PC 1 and PC 2 (A) with segments connecting points from each municipality. Using identical axis values, we plotted forest and urban individuals from each municipality individually (B) to better visualize variation across municipalities. The first between group PC axis described 12.0% of the variation, while bgPC 2 captured 6.3%. Lastly, we projected minimum and maximum shape extremes for both bgPC axes (C). These illustrations represent shapes two standard deviations above or below the overall mean shape along each bgPC axis.

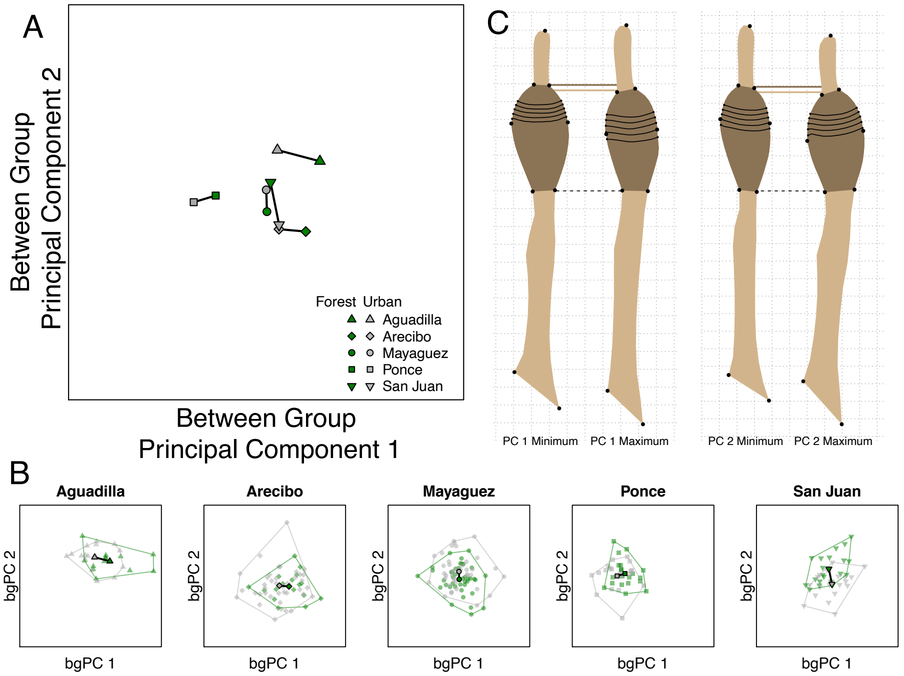

<u>Figure S6</u>

We compared toepad width versus length for urban and forest lizards. In each scatter plot, we illustrate pad width versus length in millimeters between urban (gray) and forest (green) lizards from each municipality. The slopes of the fitted lines forced through the origin represent the mean ratios for urban and forest lizards. Note the grey urban line's location often below the green forest line suggesting urban lizards have a lower width/length ratio, suggesting urban lizards are gaining toepad area via larger increases in length than width. Black lines connect each intra-municipality mean forest and urban values.

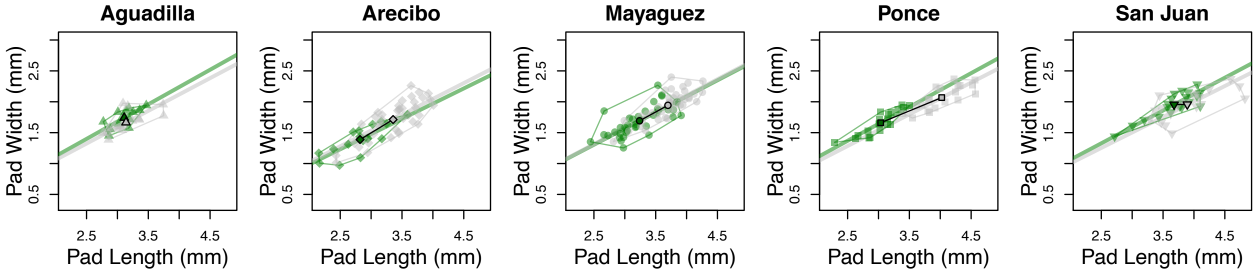

We also generated municipality-specific scatter plots of toepad area vs lamellae count and toepad area vs lamellae height, with forest lizards in green and urban lizards in grey. We also included forest and urban population means with black line segments connecting the means to better highlight the population differences.

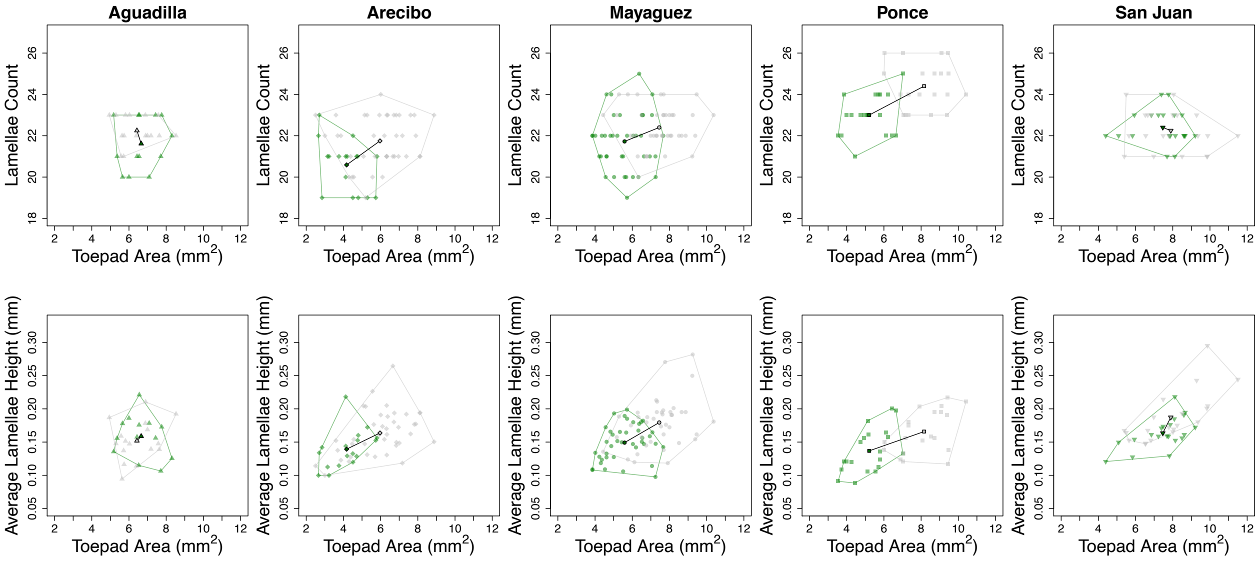

We lastly generated municipality-specific scatter plots of total toe length and pad length for forest and urban lizards using green and grey points with best-fit lines forced through the origin. We also included black line segments connecting forest and urban means to highlight the difference in ratios between urban and forest populations.

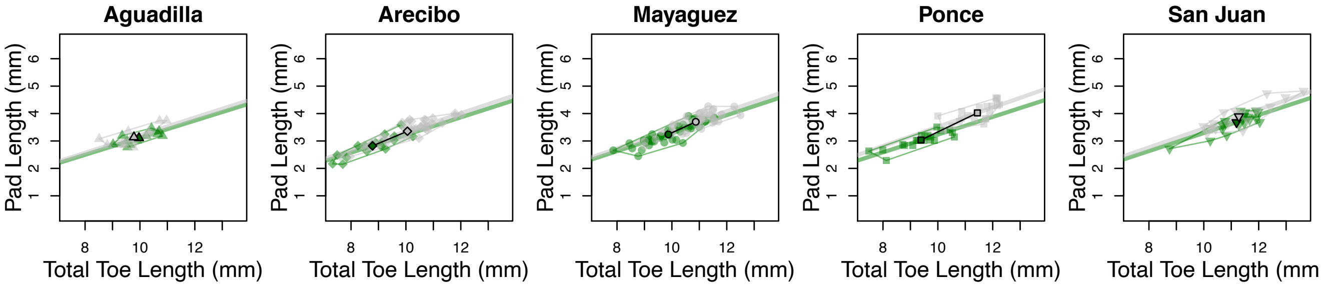

<u>Figure S7</u>

We investigated the relationship between toepad length and the number of lamellae (top plot) and lamellae height (lower plot) using green and grey points to represent forest and urban lizards. Both toepad length and lamellae height were natural log transformed for analyses, while lamellae count was square root transformed. The lines represent linear model fits to our transformed data, hence why we used the non-periodic axes labels on the plots.

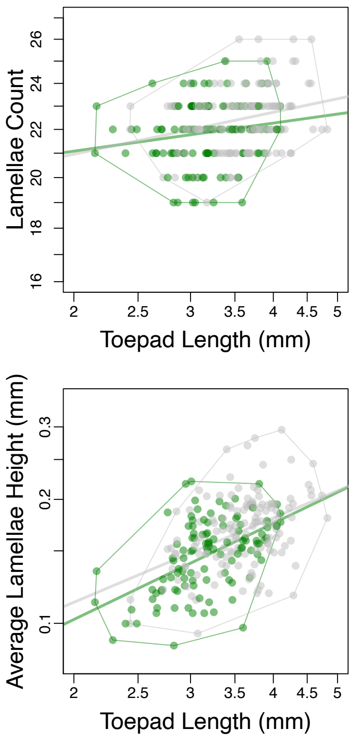

Methodological Analyses

Methods

Given our novel approach of applying geometric morphometrics to investigate adhesive toepad shape, we conducted analyses to further investigate the use of this method. We compared different approaches to estimate toepad area, the quantified toe pad allometry, and investigated how specimen size was captured by our PCA.

Considering approaches to measure toepad area, we investigated the relationship between toepad area (measured as the area of the enlarged portion of pad and toe to nearest phalangeal joint below the pad) and variables of size extracted from our geometric morphometrics data, specifically centroid size, which is the square root of the sum of the squared distances of each landmark from the specimen's centroid (i.e. center), estimated by the alignment function, "gpagen", in *geomorph*. We hypothesized that, instead of only acting as a proxy for area, landmark data could be used to directly measure area, eliminating the need to measure it independently. Using landmarks 3, 4, 5, 6, 7, 8 and the semilandmark curves connecting them (see Fig. 2), we calculated the area enclosed by these landmarks using the "polyarea" function in the *pracma* R package. To compare our measurement of toepad area from ImageJ to centroid size and our landmark polygon area, we natural-log transformed all three variables and used separate analyses of variance to determine how toepad area from ImageJ is related to centroid size and how tightly they were correlated using Pearson's R as well how toepad area from ImageJ is related to our landmark polygon area and how tightly they were correlated also using Pearson's R (Fig S8).

While our area measurements (from ImageJ or our landmark polygon) are intuitive measurements, centroid size can be affected by shape. In other words, two individuals could have the area, but if they are shaped differently, they will likely have different centroid sizes. To examine this, we extracted residual values from our centroid size~area analyses of variance to get an estimate of centroid size after the effect of area had been removed leaving only the effect of shape. We correlated these residual values with the ratio of toe width over length, using a linear model to test for slope and intercept values significantly different than zero. If centroid size is not affected by shape, all of the centroid size residuals should be near zero and should not correlate with a measurement of shape (i.e. toe width/length). A positive correlation would suggest wider pads artificially inflate centroid size. Conversely, a negative correlation would suggest longer pads inflate centroid size.

After viewing these results, we were interested in measuring toe pad area in a way that was more complimentary to our geometric morphometric landmark locations. We returned to our original toepad images and measured toepad area for a subset of specimens again using ImageJ, randomly selecting 66 individuals across our previous ImageJ area measurements, although now limiting our measurement of toepad area to only the dilated area to the toe, not including the area down to the phalangeal joint (measured by author #3, using embedded scale bars or image resolution to set scale). This more conservative measurement of toepad area is more consistent with the locations of our 3rd and 4th landmarks, and we hypothesized it would make a better comparison to the area outlined by our landmarks 3-8. We compared our landmark-based polygon measurements of area to our new measurements of area from ImageJ, testing if these measurements are equivalent using paired t-tests to test for similar means (Fig. S8) and calculating slope, intercept and Pearson's R values.

We were also interested in aspects of shape that may be related to specimen size (i.e. allometry). In this case, we considered specimen size defined as toepad area, measured down to the nearest phalangeal joint below the pad, and as centroid size. We used the "procD.lm" function in *geomorph* to conduct a type-2 analysis of variance to test for significant correlations between shape and natural log transformed area and natural log transformed centroid size, including the interaction between area and centroid size. We then used the "shape.predictor" function to visualize significant differences in shape (Fig. S8). We also investigated if specimen size is correlated with principal component axes, as they are often assumed to be. After again calculating PC axes using the "gm.prcomp" function in the *geomorph* package (see Fig. 4), we conducted Pearson's correlation analyses, testing if different variables of size are significantly correlated with PC axis 1-3. We considered natural log transformed SVL, natural log transformed toepad area (measured to the nearest phalangeal joint), and natural log-transformed centroid size (Fig. S9).

<u>Methodological Results</u>

We conducted analyses to validate the use of geometric morphometrics to study toe and toepad morphology. We found both our landmark-based measurement of area and centroid size to be strongly correlated with toepad area measured from ImageJ (Fig. S8). However, we caution that due to the differences in the units of centroid size (mm) and area (mm^2) and how centroid size is calculated, area and centroid size are not interchangeable. In addition, we extracted residuals our ImageJ area

correlation with centroid size and compared them to the ratio of pad width over length (Fig S8 plot B insert). Specimens with a low toe width/length ratio were long and skinny, whereas specimens with higher ratios were shorter and wider. We found a significant negative relationship between centroid size residuals and toe width/length (slope = -1.49, slope p value <0.001, intercept = 0.22, intercept p value < 0.001). This result suggests centroid size and its relationship with area is strongly affected by sample shape, with shorter/wider specimens having smaller centroid sizes, after accounting for area. In addition, we found our measurement of area from ImageJ (which included the area to the joint below the pad) to be strongly correlated with area measured from our landmarks (Pearson's R: 0.97, p < 0.001; Fig. S8 B). Although these two variables are tightly correlated, they are not equivalent: area measured from our landmarks was approximately 78% of the area estimated from ImageJ.

The relationship between shape and centroid size becomes clear when how centroid size is calculated is considered. Centroid size is a summarized measurement of how far away landmarks are from the center of a specimen, but during the calculation of centroid size, these distances are squared, disproportionately increasing already large values. Keeping area constant across a set of idealized models, samples with the smallest centroid sizes would be those that most approach a sphere, with all of their landmarks being as close to the center as possible. Alternatively, samples with some landmarks near the center and some landmarks far away from the center (either star shaped, or in our case long and skinny toepads), even with the same area, these specimens will have a larger centroid size.

Given the above strong correlations, yet significant differences between toepad area as defined to include the area to the joint below the pad and the area captured by our landmark polygon, we re-measured a subset of individuals in ImageJ, only measuring the dilated area of the pad. While we found that while area measured by the two approaches were significantly different using a paired t-test (t = -15.6, df = 65, p < 0.001), Pearson's R analyses (Pearson's R: 0.993, Fig. S8 C) suggest they were strongly correlated and nearly equivalent (slope of 1.01, Y-intercept of 0.25). Our conservative ImageJ mean area was 4.56mm^2 ± 0.30, while the mean area calculated from our landmarks was 4.85mm^2 ± 0.31 (± 95% CI). The average difference between remeasured individuals was 0.291mm^2. The similarity of our estimated slope value to 1.0, the very high Pearson's R value suggesting a tight correlation, the very small mean difference, and the similarly between the difference in means and our estimated Y-intercept suggests the differences between these datasets may be a result of investigator differences, with author #1 having placed the landmarks while author #3 measured area via ImageJ. After

calculating the difference between each of our landmark polygon area measurements and the same individual's conservative ImageJ area measurement, we plotted these differences against area to better visualize the differences in these measurements of area, also fitting a linear model line (embedded plot Fig. S8 D). Our landmark polygon measurements of area are consistently roughly 0.25mm^2 larger than our ImageJ measurements of area, and only subtly increased with area, again likely suggesting investigator differences in judging toepad boundaries.

We also investigated the influence size may be having on our shape data. We considered two definitions of specimen size; natural log transformed toepad area measured to the phalangeal joint proximal to the pad and natural log transformed centroid size. We found that area and centroid size were both significantly related to shape (Procrustes LM, $p = 0.001$ for both) while the interaction was not ($p = 0.17$). Given centroid size's previously described relationship with shape and size, the significant interaction between area and centroid size is not surprising. Specimens with larger toepad area and larger centroid size both had enlarged pad areas, shorter proximal toe sections, and distally shifted lamellae 5-10 (Fig. S8 E).

<u>Figure S8</u>

To compare landmark-based measurements of toepad size to area measured from ImageJ, we compared centroid size and three different measurements of toepad area (A); area measured in ImageJ descending to the proximal toe joint, polygon area using geometric morphometrics landmarks 3-8, and area measured in ImageJ only including the dilated area of the pad.

We compared ImageJ area (measured to the proximal joint) to centroid size to (B), extracting the residuals from our centroid size correlation and comparing these to the ratio of pad width/length (B insert), to visualize how centroid size is correlated with toepad shape after area has been removed, with the dashed line representing the $y = 0$ line and the solid line representing our linear model fit.

We also compared area measured in ImageJ descending to the proximal toe joint to the area enclosed by landmarks 3-8 and connected semilandmarks (C). We included a dashed line ($x=y$) and a solid line representing our linear model fit. Pearson's R estimates and an analysis of variance p value are included in the upper left of both plots.

We also compared a subset of toepad area measurements collected from ImageJ (measuring only the dilated pad area) to toepad area estimated using a polygon around our geometric morphometric landmarks (D). The dotted line represents our expected relationship (X = Y) and the solid line is the fitted line to our data. We included estimates of slope and y-intercept, Pearson's R estimates, and paired t-test values. In the inserted plot, we illustrate differences between our two measures compared to area to better understand how these two measurements vary with a dotted line at y=0 and the solid line representing a linear model fit.

Lastly, we plotted projected shape differences (E). We considered how shape varied with centroid size (left pair) and toepad area measured from ImageJ to the proximal joint (right pair), highlighting the effect of specimen size on shape in our data.

Geometric Morphometrics Reveal Shape Differences in the Toes of Urban Lizards

Supplemental Figures and Tables

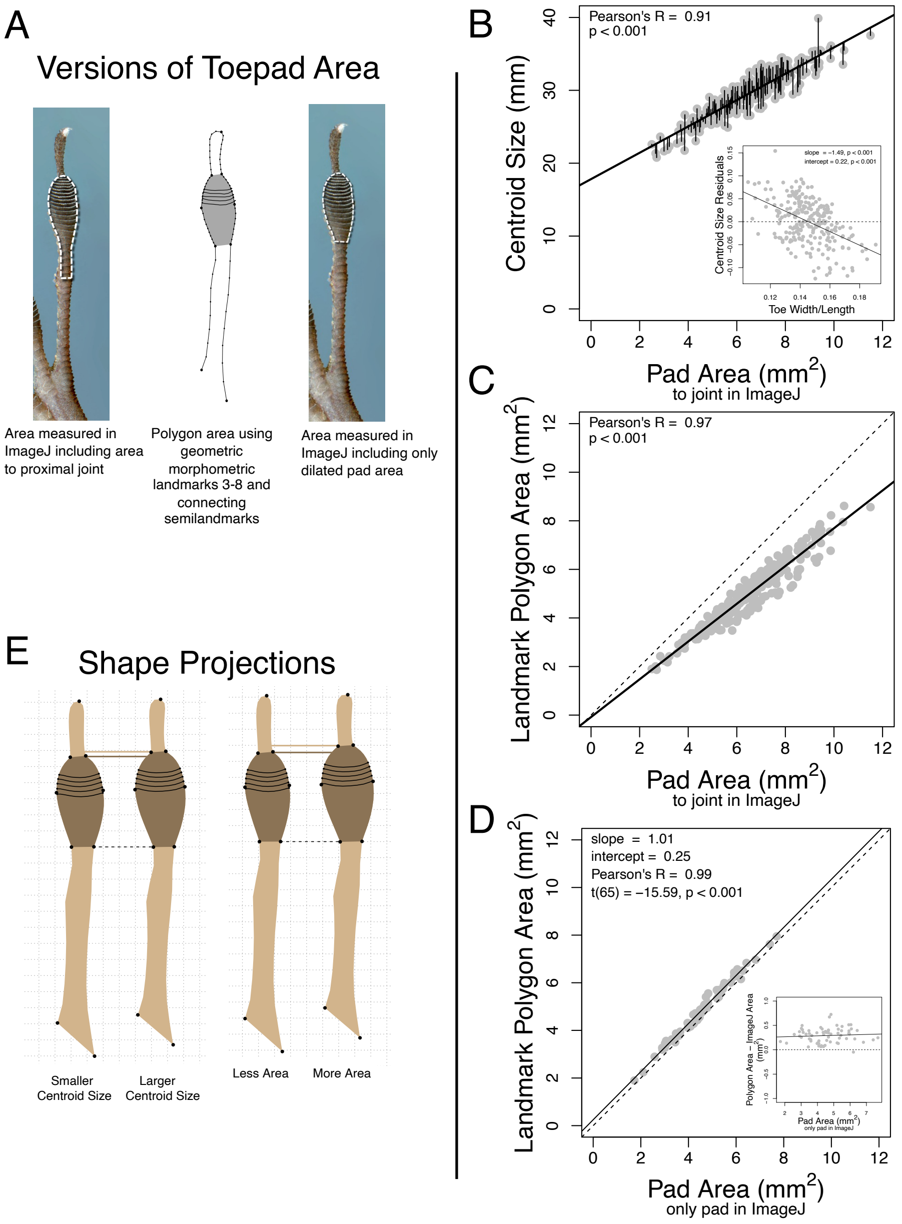

In addition to investigating the relationship between specimen size and shape, we also explored how size is captured by our first three principal component axes (Fig. S9). We found natural log transformed SVL to not significantly correlate with PC 1 (Pearson's R = 0.018, p = 0.77), significantly negatively correlate with PC 2 (Pearson's R = -0.25, p < 0.001) and significantly positively correlate with PC 3 (Pearson's R = 0.41, p < 0.001). Similarly, natural log toepad area measured to the nearest phalangeal joint below the toepad also did not significantly correlate with PC1 (p = 0.13, Pearson's R = 0.10), negatively correlated with PC 2 (p < 0.001, Pearson's R = -0.26), and positively correlated with PC 3 (p < 0.001, Pearson's R = 0.52). Lastly, natural log centroid size followed a similar pattern, with a non-significant relationship with PC 1 (p = 0.11, R = 0.10), a significant negative relationship with PC 2 (p < 0.001, R = -0.28), and a significant positive relationship with PC 3 (p < 0.001, R = 0.37). Interestingly, it appears PC1 did not capture specimen size no matter its defined (SVL, area, or centroid). Considering our projected toe shapes along our PC axes (Fig. 4 and S9), with PC 1 primarily capturing toe curvature and some aspects of toe and toepad internal proportions, PC 2 capturing lamellae morphology and some aspects of toepad length, and PC 3 most clearly capturing relative toepad size, these results appear consistent.

<u>Figure S9</u>

We plotted the relationships between our first three PC axes and body size, represented by natural log SVL (left column of plots), natural log toepad area measured to the nearest phalangeal joint below the toepad (center column of plots) and natural log centroid size (right column of plots). We also included our estimated trend line of each fitted linear model (solid black lines) with 95% confidence intervals (dashed lines), Pearson's R and p values (upper left of each plot). Note the nonlinear aspect of our x axes axes due to their transformations prior to analyses. The PC score are often difficult to interpret, and so these values were omitted from our y-axis, although for each row of plots, the y-axes are identical. For each PC axes, we also included estimated extreme shape projections (reprinted from Fig. 4) to better highlight the morphological variation captured by each PC axis.

Geometric Morphometrics Reveal Shape Differences in the Toes of Urban Lizards

Supplemental Figures and Tables

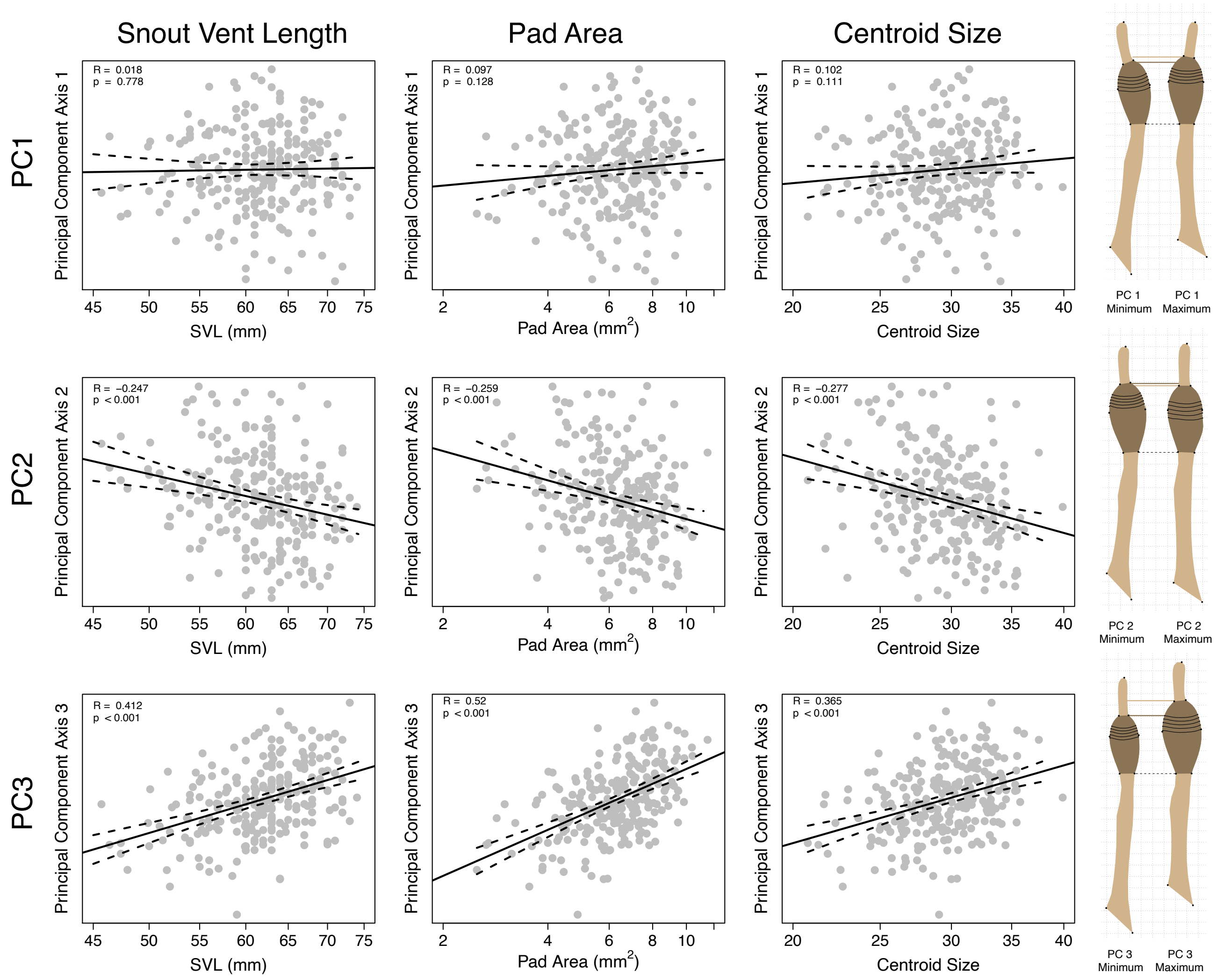

Work Cited

Bookstein, F.L. (2019) Pathologies of between-groups principal components analysis in geometric morphometrics. *Evolutionary Biology,* **46,** 271-302.
